# Clinical outcomes, anticoagulation practice patterns, and quality of life after cancer-associated pulmonary embolism: secondary outcomes of the Hydra randomized clinical trial

**DOI:** 10.1016/j.rpth.2026.107018

**Published:** 2026-10-13

**Authors:** Bram Akerboom, Emily S.L. Martens, Britta Buechler, Luca Valerio, Stavros Konstantinides, Michiel Coppens, Francis Couturaud, Yordi P.A. van Dooren, Marco J.J.H. Grootenboers, René van der Griend, David Jiménez, Jenneke Leentjens, Roberto Pola, Rosa Talerico, Tobias Tritschler, Hugo ten Cate, Karly Hamulýak, Thijs E. van Mens, Frederikus A. Klok, Menno V. Huisman

**Affiliations:** 1 Department of Medicine – Thrombosis and Hemostasis, Leiden University Medical Center, Leiden, the Netherlands; 2 Center for Thrombosis and Hemostasis, University Hospital of the Johannes Gutenberg University Mainz, Germany; 3 Department of Vascular Medicine, Amsterdam UMC, University of Amsterdam, Amsterdam, The Netherlands; Amsterdam Cardiovascular Sciences, Pulmonary Hypertension & Critical Care, Amsterdam, The Netherlands; 4 Department of Medicine and Pulmonology, CHU Brest, INSERM U1304-GETBO, Brest University, Brest, France; 5 Department of Pulmonology, Groene Hart Hospital, Gouda, The Netherlands; 6 Department of Pulmonology, Amphia Hospital, Breda, The Netherlands; 7 Department of Medicine, Diakonessenhuis Utrecht, Utrecht, The Netherlands; 8 Respiratory Department, Ramón y Cajal Hospital (IRYCIS), Madrid, Spain; Department of Medicine, Universidad de Alcalá (IRYCIS), Madrid, Spain; CIBER de Enfermedades respiratorias (CIBERES), Madrid, Spain; 9 Department of Medicine, Radboud Institute for Health Sciences, Radboud University Medical Centre, Nijmegen, The Netherlands; 10 Percorso Trombosi, Dipartimento di Scienze dell’Invecchiamento, Ortopediche e Reumatologiche, Fondazione Policlinico Universitario A. Gemelli IRCCS, Università Cattolica del Sacro Cuore, Rome, Italy; 11 Department of General Internal Medicine, Inselspital, Bern University Hospital, University of Bern, Bern, Switzerland; 12 Cardiovascular Research Institute Maastricht, Maastricht University, Maastricht, the Netherlands; Thrombosis Expertise Center, Heart+Vascular Center, Maastricht University Medical Center, Maastricht, the Netherlands; 13 Department of Internal Medicine, Maastricht University Medical Center (Maastricht UMC+), Maastricht, The Netherlands

**Keywords:** anticoagulants, hemorrhage, neoplasm, pulmonary embolism, venous thromboembolism

## Abstract

**Background:**

Despite its high prevalence, practice-based data on management and outcomes after cancer-associated pulmonary embolism (PE) are scarce, and no studies have investigated quality of life.

**Objectives:**

We aimed to evaluate clinical outcomes, anticoagulation practice patterns, and quality of life after cancer-associated PE.

**Methods:**

The Hydra randomized clinical trial assessed the safety of the YEARS algorithm versus computed tomography pulmonary angiography only to exclude acute PE in patients with cancer. Patients with PE at baseline or during 90-day follow-up were included in this study. Outcomes were recurrent venous thromboembolism, major bleeding, all-cause mortality, anticoagulation practice patterns, and quality of life as assessed by the Pulmonary Embolism Quality of Life (PEmb-QoL) questionnaire. PEmb-QoL scores were compared with those for the general PE population reported in the FOCUS study. Lower scores indicate better QoL.

**Results:**

A total of 125 patients were included, of which 103 (82%) had PE at baseline, and 22 (18%) developed PE during follow-up. The 90-day cumulative incidence of recurrent venous thromboembolism and major bleeding was 2.5% (95% CI 0.67%-6.6%) and 2.5% (95% CI 0.68%-6.7%), respectively. All-cause mortality at 90 days was 32% (95% CI 23%-40%). Two-thirds of patients continued anticoagulation until less than 24 hours before death, and the median time between stopping anticoagulation and death was 10 days (IQR, 4-14). The median 90-day PEmb-QoL score was 33% (IQR, 13-51) versus 20% (IQR, 10-40) reported in the FOCUS study.

**Conclusion:**

Patients with cancer-associated PE suffer from high mortality, high morbidity, and impaired quality of life. Anticoagulation therapy is frequently continued until the last days of life.

## Essentials

• Data on management and quality of life after cancer-associated pulmonary embolism are scarce.
• Data from the Hydra study, which assessed the YEARS algorithm in patients with cancer, were used.
• Recurrent thrombosis, bleeding, and mortality were frequent.
• Anticoagulation was frequently continued until death and quality of life after pulmonary embolism was impaired.

## 1 Introduction

Patients with cancer face an elevated risk of venous thromboembolism (VTE), which has been increasing over the past decades [1]. Although anticoagulation is standard care for cancer-associated VTE, it is associated with a high risk of bleeding, and recurrent VTE still occurs often in this group of patients [2], [3], [4]. This is especially relevant for patients with end-stage cancer, who have been systematically excluded from most clinical trials on anticoagulation treatment for pulmonary embolism (PE) and also other conditions requiring anticoagulation, such as atrial fibrillation and deep vein thrombosis [5], [6], [7].

Consequently, physicians lack clear, evidence-based guidance on the risks and benefits of anticoagulation during end-of-life care in patients with cancer. To address this uncertainty, the Europe-wide Serenity Project was launched. Its goal is to develop and validate a clinical decision-support tool to guide discussions about continuing or discontinuing anticoagulation as patients with cancer approach the end of life [7]. As part of this effort, several descriptive studies have analyzed European nationwide databases to better understand current antithrombotic treatment patterns during end-of-life care [8], [9], [10].

Beyond clinical outcomes, PE is also known to impair quality of life [11], [12], [13], [14], and this impact may be even more pronounced in patients with cancer due to their higher vulnerability and poorer overall prognosis. Studies on quality of life after PE have included only a minority of patients with cancer, limiting the generalizability of findings to this high-risk group [11], [12], [13]. Therefore, a dedicated study assessing quality of life after cancer-associated PE is needed.

In the present paper, we report on clinical outcomes, anticoagulation practice patterns, and quality of life after cancer-associated PE. Furthermore, the current study aims to support the Serenity Project by providing additional data on anticoagulation-related outcomes and treatment complications during end of life in patients with cancer-associated PE.

## 2 Methods

### 2.1 Hydra study

The design and methods of the Hydra study have previously been published (International Clinical Trials Platform registry number: NL-OMON52383) [15]. Briefly, the Hydra study was a randomized, investigator-initiated, noninferiority trial with blinded central outcome adjudication, involving 21 hospitals in 6 countries (the Netherlands, Italy, Switzerland, France, Spain, and Belgium). From August 22, 2019, to May 23, 2025, 698 patients were recruited from emergency departments, outpatient clinics, and during hospitalization. The trial showed that in patients with cancer, a diagnostic strategy using the YEARS algorithm was as safe as computed tomography pulmonary angiography (CTPA) only for excluding acute PE, while obviating the need to perform a CTPA in 22% of patients [16]. All participants provided written informed consent before randomization, and the study was approved by the institutional review board of each participating center. This paper describes the predefined secondary outcomes of the Hydra study.

Patients 18 years or older with active cancer and a clinical suspicion of acute PE were eligible for inclusion. Active cancer was defined as a cancer diagnosis confirmed by histology or strongly suspected by a clinician within 6 months before study inclusion; ongoing cancer treatment at the time of inclusion; cancer treatment received within 6 months before randomization; or the presence of metastatic disease. Patients were excluded if they had therapeutic anticoagulation for indications other than acute PE, had prior participation in the study, had PE diagnostics initiated because of a suggestion of PE on a previously performed radiological scan, were hemodynamically unstable, had contraindications to CTPA, were participating in a concurrent study on thromboprophylaxis, or if they had an expected life expectancy of less than 3 months.

For this analysis, we included all patients with acute symptomatic or incidental PE diagnosed at baseline or during follow-up, or PE-related death during follow-up. PE was defined as an intraluminal filling defect in a subsegmental or more proximal pulmonary artery on CTPA or a mismatch between ventilation and perfusion with a perfusion defect at a segmental or more proximal level on a ventilation-perfusion scan. PE-related death was defined as any death adjudicated as either certain PE-related or possible PE-related (ie, unexplained) death adjudicated according to the International Society on Thrombosis and Haemostasis (ISTH) definition for PE-related death [15], [17].

### 2.2 Outcomes

Patients were instructed to contact study personnel or the emergency department in case of signs or symptoms suggestive of VTE or major bleeding (MB) during follow-up. Every patient also received a follow-up telephone call at 90 days (±7 days) to ask about signs and symptoms suggestive of recurrent VTE or MB and to evaluate anticoagulation practice patterns. If the patient did not answer, the general practitioner and/or primary cancer physician were contacted.

All suspected recurrent VTE and MB during follow-up were adjudicated by HtC and KH. Disagreements were resolved by discussion, and if necessary, a third thrombosis expert was consulted. VTE was incidental if it was detected by means of imaging tests performed for reasons other than clinical suspicion of VTE.

Recurrent VTE was defined as [18]:

• a new intraluminal filling defect or an extension of an existing defect; or
• an area of noncompressibility in a venous segment where compression had been normal; or
• a significant increase (>4 mm) in vein diameter of a previous noncompressible segment upon compression ultrasound.

MB was defined according to the ISTH definition [19]:

fatal; and/or associated with any of the following:

• a fall in hemoglobin level of >2 g/dL (ie, >1.24 mmol/L); or
• documented transfusion of >2 units of packed red blood cells or whole blood; or
• involvement of a critical area or organ, such as intracranial, spinal, ocular, pericardial, articular, intramuscular with compartment syndrome, or retroperitoneal.

Additionally, 90 days (±7 days) after inclusion in the Hydra trial, all patients with PE at baseline or during follow-up were asked to complete the Pulmonary Embolism Quality of Life (PEmb-QoL) questionnaire. The PEmb-QoL questionnaire was initially developed and validated in Dutch and English to specifically measure PE-related quality of life [20], [21], and was subsequently translated and validated in French [22] and German [23], and has also been translated into Spanish and Italian. The questionnaire assesses health-related quality of life in six dimensions: frequency of complaints, limitation in activities of daily living (ADL), work-related problems, social limitations, intensity of complaints, and emotional complaints. Percentage scores (0-100) were calculated for each dimension as well as an overall score, with a higher score indicating a worse quality of life. PEmb-QoL is one of the central instruments from the International Consortium for Health Outcomes Measurement standard set of outcome measures for patients with VTE [24]. As an exploratory analysis, the scores were graphically compared with the 3-month PEmb-QoL scores after PE in the FOCUS study from the population of all-comers [12].

### 2.3 Focus study

The FOCUS study (German Clinical Trials Registry number DRKS00005939) prospectively included 1098 consecutive unselected patients with a confirmed diagnosis of acute symptomatic PE between September 2014 and October 2018 at 17 large-volume centers across Germany. Patients were followed up over a 2-year period and were treated according to the standard of care at the participating centers without any specific mandates from the study protocol [12], [25]. The current analysis included patients who had completed at least one item in all numeric dimensions of the 40-item PEmb-QoL questionnaire at the 3-month visit.

### 2.4 Statistical analysis

Patient characteristics and anticoagulation practice patterns were described using means with SD or medians with IQR as appropriate for continuous variables and counts with percentages for categorical variables. The Kaplan–Meier estimator was used to estimate 30-day and 90-day all-cause mortality. The incidence of recurrent VTE and MB was estimated using the cumulative incidence function in which death was considered a competing risk. The date of PE diagnosis was used as the index date. The PEmb-QoL scores were calculated for each dimension by summing up the item values divided by the number of items. If a question was missing, the concurrent dimension was not calculated for that patient. The overall score was calculated by averaging the score for each dimension. Anticoagulation practice patterns were only described for patients with confirmed PE on imaging.

No imputation for missing data was performed. We used R version 4.2.1 (R Foundation for Statistical Computing, Vienna, Austria) for all statistical analyses. Cumulative incidences were estimated using the *tidycmprsk* package.

## 3 Results

### 3.1 Study population

A total of 125 patients with cancer-associated PE were included, of whom 103 (82%) had acute PE diagnosed at baseline and 22 (18%) during follow-up, of which 9 were symptomatic PE, 3 were incidental (ie, unsuspected) PE, and 10 were PE-related deaths. Baseline characteristics are summarized in Table. The median age at baseline was 66 years (IQR, 59-73), and 72 (58%) patients were female. A history of VTE was reported in 10 (8.0%) patients, and 19 (16%) had undergone recent surgery (<1 month). Regarding the largest vessel involved, PE was central in 35 (30%) patients, segmental in 54 (47%), and subsegmental in 26 (23%). Of the patients with PE at baseline, 22 (21%) were discharged within 24 hours. No patients were lost to follow-up; data on anticoagulation management during follow-up were missing for 2 patients.

**Table:** Baseline characteristics

Number of patients	125
Age (years)	66 (59,73)
Female sex	72 (58)
Body mass index	26 (23,30)
Smoking history	
No	60 (48)
Former	50 (40)
Current	15 (12)
Antiplatelet therapy^a^	19 (15)
History of venous thromboembolism	10 (8.0)
Recent surgery	19 (16)
Symptomatic PE	122 (98)
Incidental PE	3.0 (25)
Largest vessel involved	
Central	35 (30)
Segmental	54 (47)
Subsegmental	26 (23)
Primary cancer site	
Breast	17 (14)
Lung	34 (27)
Upper gastrointestinal tract	8.0 (6.4)
Lower gastrointestinal tract	13 (10)
Genitourinary tract	31 (25)
Brain	4.0 (3.2)
Hematological	11 (8.8)
Melanoma skin cancer	3.0 (2.4)
Other	9.0 (7.2)
Anticancer therapy in previous 6 months	
Chemotherapy	71 (57)
Surgery	25 (20)
Radiation therapy	18 (14)
Immunotherapy	35 (28)
Hormone therapy	7.0 (5.6)
No anticancer therapy	15 (12)
Cancer stage	
Localized	25 (20)
Regional	25 (20)
Distant metastases	67 (54)
Comorbidities	
Hypertension	49 (39)
Diabetes mellitus	15 (12)
COPD	18 (14)
Ischemic heart disease	7.0 (5.6)
Stroke	8.0 (6.4)
Heart failure	2.0 (1.6)
Rheumatic or other autoimmune disease	5.0 (4.0)
Presenting symptoms	
Dyspnea	99 (85)
Chest pain	57 (49)
Syncope	10 (8.8)
Hemoptysis	9.0 (7.8)
Palpitations	13 (12)

Data are n (%), or median (IQR).

COPD, chronic obstructive pulmonary disease; PE, pulmonary embolism.

a No data on changes in antiplatelet therapy after baseline are available.

### 3.2 Anticoagulation practice patterns

Of the 115 patients with symptomatic or incidental PE diagnosed on imaging, 64 (56%) patients were treated with a direct oral anticoagulant (DOAC). Forty-three (37%) patients were initially treated with low-molecular-weight heparin (LMWH), of which 13 (33%) remained on LMWH at the end of follow-up, 25 (64%) were transitioned to a DOAC, 1 (2.6%) was transitioned to a vitamin K antagonist, 3 (7.0%) patients discontinued anticoagulation, and for 1 (2.6%) patient data were missing. The median time to transition from LMWH to DOAC was 6 (IQR, 3-19) days. Four (3.5%) patients were treated with unfractionated heparin, and all were transitioned to a DOAC by the end of follow-up. Two (1.7%) patients were treated with a vitamin K antagonist. Two (1.7%) patients had a subsegmental PE and a high risk of bleeding and therefore did not receive anticoagulation therapy. Neither experienced recurrent VTE or MB or died during the follow-up period. Anticoagulation management during initial diagnosis of PE and at the end of follow-up is illustrated in Figure 1.

**Figure 1:**
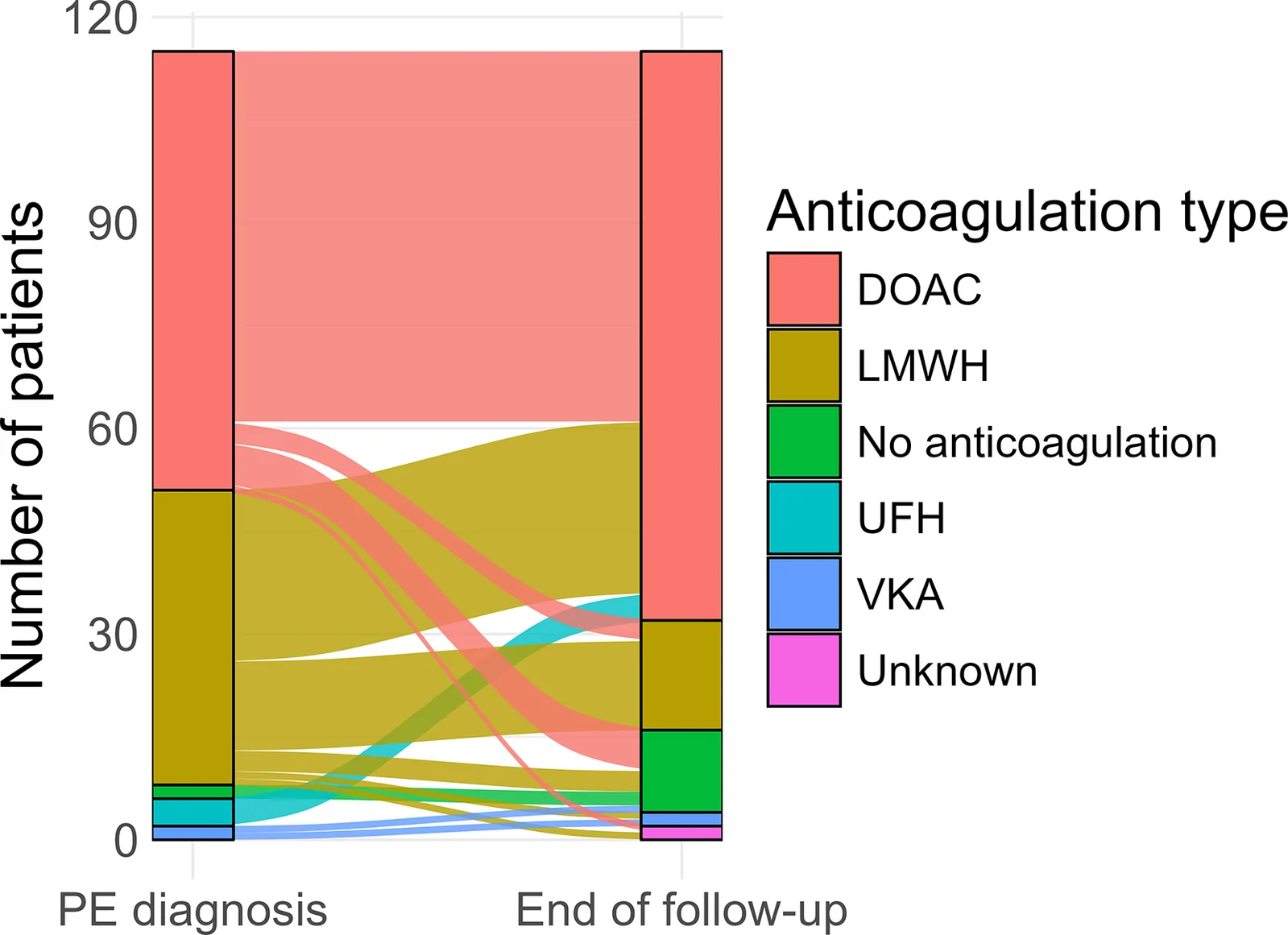
Alluvial diagram visualizing anticoagulation changes from PE diagnosis to end of follow-up. DOAC, direct oral anticoagulant; LMWH, low-molecular-weight heparin; UFH, unfractionated heparin, VKA, vitamin K antagonist.

### 3.3 Clinical outcomes and anticoagulation management during end of life

After cancer-associated PE, 39 patients died during follow-up, of whom 10 died because of PE. The 30- and 90-day all-cause mortality were 22% (95% CI, 14%-29%) and 32% (95% CI, 23%-40%), respectively (Figure 2). Anticoagulation was continued until at least 24 hours prior to death in 70% (n = 21, 95% CI, 50%-85%) of patients who initially started anticoagulation. Reasons for stopping anticoagulation were end-of-life care (n = 6), bleeding complications (n = 2), and severe thrombocytopenia (n = 1). If anticoagulation was stopped more than 24 hours prior to death, the median number of days between stopping anticoagulation and death was 10 (IQR, 4-14). For 1 additional patient, anticoagulation was stopped because of end-of-life care 81 days after PE diagnosis. This patient was still alive at the end of the follow-up period.

**Figure 2:**
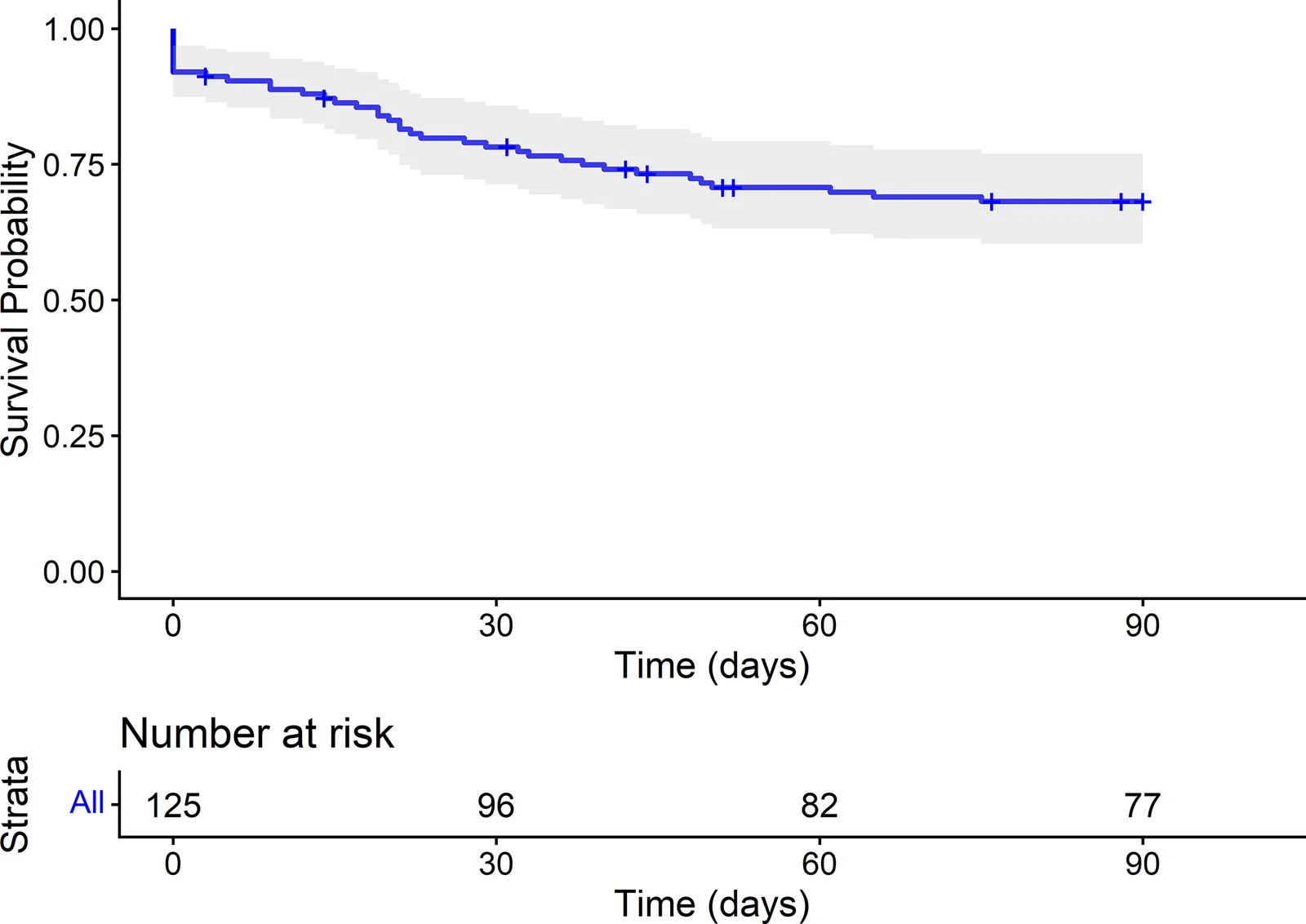
Cumulative survival probability after cancer-associated PE with 95% confidence interval.

Three patients had recurrent VTE, all of which were nonfatal PE. Two were on DOAC during the VTE recurrence and were transferred to LMWH; one was on a DOAC, which was discontinued by the patient without consulting a physician and restarted on VTE recurrence. Three patients had a (nonfatal) MB during follow-up, all of which were during treatment with LMWH. One had an adrenal hemorrhage, 1 had an intramuscular hemorrhage of the right upper leg, and 1 had an intraabdominal bleed. Two patients stopped anticoagulation therapy indefinitely, and the third patient was transferred to a DOAC. The cumulative incidence of recurrent VTE and MB at 90 days was 2.5% (95% CI, 0.67%-6.6%) and 2.5% (95% CI, 0.68%-6.7%), respectively.

### 3.4 Quality of life

A total of 85 patients with acute PE at baseline or during follow-up did not die and were therefore eligible to complete the PEmb-QoL questionnaire. Of these, 67 (79%) patients completed the questionnaire. Reasons for not completing the questionnaire were: not sent by study personnel (n = 4), patient refused (n = 5), no response (n = 6), end-of-life care (n = 1), and unknown (n = 2). The results of the PEmb-QoL questionnaire for each dimension can be found in Figure 3. The median overall PEmb-QoL score was 33% (IQR, 13-51) versus a median score of 20% (IQR, 10-40) in the FOCUS study. For the Hydra study, the median scores for each individual dimension were 12% for frequency of complaints, 50% for limitation in ADL, 75% for work-related problems, 25% for social limitations, 20% for intensity of complaints, and 18% for emotional complaints. For the FOCUS study, the median scores were 20% for frequency of complaints, 25% for limitation in ADL, 0% for work-related problems, 0% for social limitations, 24% for intensity of complaints, and 20% for emotional complaints.

**Figure 3:**
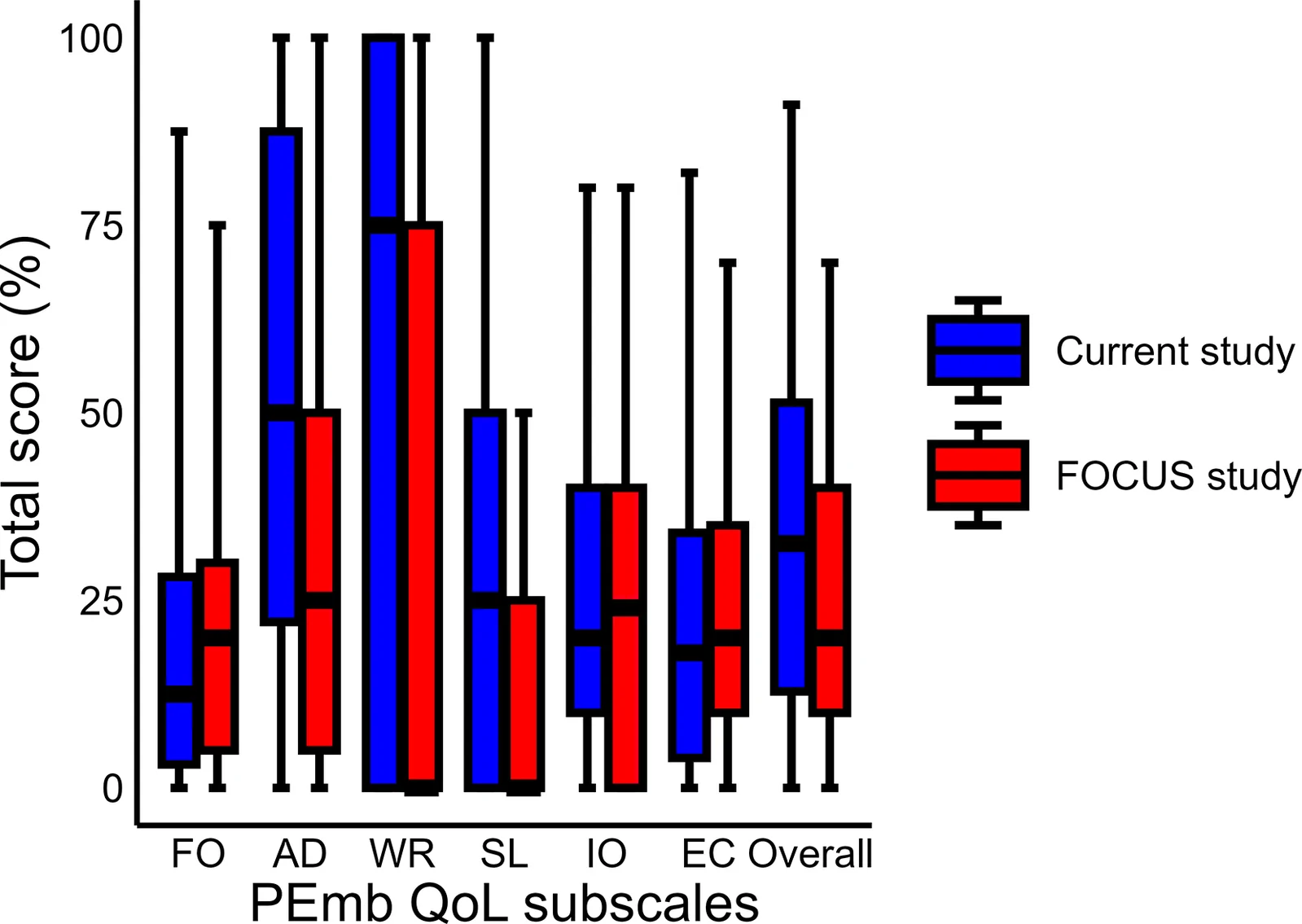
Distribution of dimension-specific and overall QoL as assessed by the PEmb-QoL score at 90 days of our study in blue and the FOCUS study by Valerio et al. [12] in red. Higher PEmb-QoL scores reflect decreased quality of life. AD, activity of daily life (ADL) limitations; EC, emotional complaints; FO, frequency of complaints; IO, Intensity of complaints; QoL, quality of life; SL, social limitations; WR, work-related problems.

## 4 Discussion

In this study, we found high mortality and morbidity in the first 90 days after cancer-associated PE. Approximately one-third of patients died, and 5% suffered either recurrent VTE or MB. For the patients who died during follow-up, the majority continued with therapeutically dosed anticoagulation up to the last days of life. Lastly, patients exhibited a noticeable impairment of their quality of life after PE.

The 90-day cumulative incidences of recurrent VTE and MB were 2.5% (n = 3, 95% CI, 0.67%-6.6%) and 2.5% (n = 3, 95% CI, 0.68%-6.7%), respectively. The incidence of VTE appears similar to the incidence reported in the Registro Informatizado de Enfermedad TromboEmbólica (RIETE) registry (3.0%) and slightly lower than the incidence reported in a post hoc analysis of the CARAVAGGIO trial (5.3%), which could possibly be explained by increased testing for VTE in the CARAVAGGIO trial environment [26]. The incidence of MB appears to be similar to the incidence reported in the RIETE registry (3.4%) and the post hoc analysis of the CARAVAGGIO trial (2.9%) [26]. Importantly, the wide confidence intervals preclude strong conclusions.

After cancer-associated PE, nearly all patients were treated with anticoagulation. The majority were treated with a DOAC, but some of the patients received LMWH. Possible reasons for LMWH therapy could be the presence of gastrointestinal or genitourinary malignancy, a perceived “high-risk” PE, or an inclusion date before the publication of the 2020 Dutch guideline that allowed for the use of DOAC as an alternative to LMWH [5], [6], [27]. Our sample size precludes formal analyses to dissect these options, and these hypotheses cannot be formally tested.

Approximately 70% of patients continued anticoagulation until less than 24 hours before death, which on first glance is similar to what is reported in the literature for cancer-associated thrombosis [28], and also for patients with cancer and an indication for anticoagulation that is not PE [8], [9], [29]. However, the median time between stopping anticoagulation and death in our study was 10 days. Since the nationwide studies from the Serenity Project use data on pharmacy dispensations to determine anticoagulation use, most of the patients who stopped anticoagulation in our study would have remained undetected in these studies [8], [9]. The real discontinuation rate in our study is therefore likely lower than in those studies, possibly because physicians are more hesitant to stop anticoagulation after a recent PE. Notably, the reasons given for discontinuation before death were similar to those reported in a post hoc analysis of the clinical trial Hokusai-VTE cancer [30]. In that study, a worsening performance status showed the strongest association with early discontinuation, consistent with “end-of-life care” being the most mentioned reason in our study.

The median overall PEmb-QoL score in our study was 33% (IQR, 13-51) versus a median score of 20% (IQR, 10-40) in the general PE population at the same follow-up time in the FOCUS study, suggesting at least a numerically worse quality of life in our study [12]. The difference in the overall score seems to be mainly driven by limitations in ADL, work, and social life. However, because of the nature of the study, we are not able to determine whether any difference is because PE has more negative effects on quality of life in patients with cancer or because of preexisting impairment caused by the cancer itself. In addition, the inclusion criteria differed considerably across the two studies. For this reason, we did not perform a formal statistical analysis to compare the PEmb-QoL scores, and our results should be interpreted as purely descriptive. Even so, the apparent difference in median overall score does not seem to exceed the minimal clinically important difference of 15% [31]. In spite of these limitations, we were still able to assess quality of life after cancer-associated PE, which is at least as impaired as in the general population with PE. It is notable that we found no clear differences in the frequency, intensity, or emotional nature of complaints. However, it remains unclear whether this reflects a true absence of differences or is due to other factors, such as a ceiling effect or patients attributing their complaints to cancer rather than pulmonary embolism.

A particular strength of our study is its prospective design and rigorous follow-up. All patients consented to the collection of their data from all health care providers, which enabled us to evaluate anticoagulation therapy during end-of-life care in the hospital, the home setting, and at the hospice. This allowed us to paint a complete picture of anticoagulation use and clinical outcomes after cancer-associated PE. Additionally, because of this rigorous follow-up, the response rate to the PEmb-QoL questionnaire was high, which allowed us to reliably assess quality of life after cancer-associated PE. Moreover, we included patients from 21 hospitals in different European countries, enhancing the generalizability of our findings.

Some limitations should be acknowledged. First, our population might be less representative because patients with a life expectancy of less than 3 months were excluded, which may partially have influenced the event rates. However, the 30-day mortality in our cohort of 19% (95% CI, 12%-25%) is very similar to the 14% (95% CI, 13%-16%) reported during the same time period in a Danish nationwide study, suggesting our study population is representative of the broader population [32]. Second, we have not collected data on antiplatelet treatment patterns during follow-up, which could have influenced the risk of bleeding in our population. Third, we only collected PEmb-QoL scores at 90 days following inclusion and are therefore unable to assess the further development of quality of life over time. Fourth, comparisons of QoL scores between our cohort and the FOCUS cohort are limited by substantial differences in population characteristics. Fifth, we did not collect any data on race and ethnicity, which limits the ability to assess generalizability, identify health disparities, and address potential biological or social factors influencing the results.

In conclusion, patients with cancer-associated PE suffer from a high mortality, a high incidence of recurrent VTE and MB, and an impaired quality of life within 90 days after diagnosis. Anticoagulation therapy is frequently continued until the last day of life.
